# Hard particle force in a soft fracture

**DOI:** 10.1038/s41598-019-40179-4

**Published:** 2019-02-25

**Authors:** Jichao Sun

**Affiliations:** 1 School of Water Resource and Environment, China University of Geosciences, Beijing, 100083 China; 2 Beijing Key Laboratory of Water Resources and Environmental Engineering, China University of Geosciences, Beijing, 100083 China; 3 Key Laboratory of Geological Information Technology, Ministry of Natural Resources of the People’s Republic of China, Beijing, 100037 China

## Abstract

The fissure patency of a rock mass is an important factor contributing towards the fluid production efficiency. Debris particles generated by the crushing of rock masses or other external forces can cause blockage or promote the smoothness of rock fractures. It is of immense theoretical and engineering value to analyze the mechanics of particles in rock fissures, especially under the compression of rock from both sides. In this study, through static analysis, the resultant force of particles in rock fissures is extruded by rock on both sides. The following conclusions are drawn: The resultant force increases first and then decrease with the increase of fissure angle and width when *x* is constant. The extreme point is at sin*θ* = *R*/(3*x*), *h* = 2 *R*(9*x*^2^ − *R*^2^)^0.5^/(9*x*) and the maximum of *F* is 8*πkR*^4^/(27*x*). Whereas, the bigger the joint roughness coefficient (*JRC*) of fissure is, the larger the average of fissure open angle is and the larger the average width is. As the *JRC* increases, the average resultant force decreases. The sharp point at the turning point of the fissure is easily broken, and the fissure width becomes larger, which makes the resultant force decrease. The analysis process expands the application prospects of the *JRC*. The results help to better understand the blockage and transport of particles in rock fissures.

## Introduction

In the context of groundwater driving, the mechanical action and mechanical mechanism of transport particles^1^ in rock fissures have attracted a significant amount of attention from researchers. The sediment particles in a fissure can be called the underground sediment. The sediment composed of soil particles from the interior of soils^2,3^, rock fissures, porous soils^4^, riverbanks and some other sources constitutes underground sediment. Silt and scour may be created during the movement of underground sands, which block and dredge fissures^5^. A particle’s clogging and scouring are related to the outflow velocity and production efficiency of underground fluid in a rock mass.

Coupled transporting research of soil particles and groundwater in soils and rock is an important research topic of interest, especially in rock fissures^6^. This field involves particle mechanics^7^, fluid mechanics, seepage mechanics, and solid mechanics. Heap and Kennedy^6^, by studying the scale-dependent permeability of fractured andesite, found that the equivalent permeability coefficient depends heavily on the initial rock permeability and the scale of interest^6^. Sun^7^ researched the permeability of particle soils under soil pressure and established the proposed model of soil particle penetration, which can be directly used to calculate the permeability coefficient. Candela, *et al*.^1^ conducted experiments on permeability and fracturing and found that permeability sensitivity to dynamic stressing increases after fracturing, which is a process that generates abundant particulate matter *in situ*.

Under the driving of the underground fluid transport, the sediment is not only affected by the pressure of the fluid but also by the crush mechanics of the rock surfaces on both sides of the fissure. A substantial amount of research in particle’s clogging and scouring in porous media has emerged^4,8^. However, few studies have been conducted on the effects of the squeeze mechanics and mechanism of particles by rock fissures on both sides. This study focuses on this lacuna and tries to understand the blockage and transport of particles in rock fissures.

**Figure 1:**
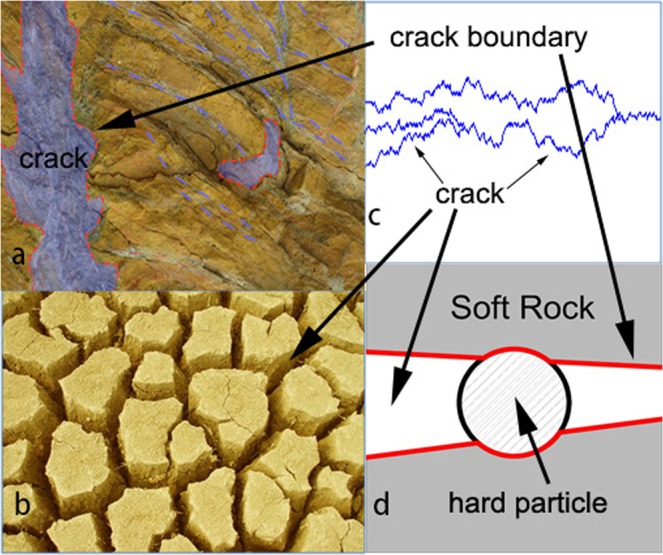
Hard particles moving in a soft rock fracture and squeezing the soils and rock. (**a**) Fissure and fracture in rock formed by the rock pressure. (**b**) Soil crack formed by evaporation. (**c**) Fracture and crack schematic diagram. (**d**) Hard particle squeezed by the soft rock from the two sides.

In the process of rock formation^9,10^, the rock is cracked (as shown in Fig. 1a), and cracks occur in the soil owing to water evaporation (as shown in Fig. 1b,c), which can considerably affect the permeability of crustal rocks^10^. Following this, in the presence of water and under the water pressure, the sediment particles enter the crack (as shown in Fig. 1d). The ground sediment is driven by groundwater, which potentially causes many hazards. If the ground sediment is fully considered and closely connected with the geological material, many geologic hazards, such as collapse of riverbanks and blockage of groundwater and oil fissures^2^, could be better handled. If the sediment particles are very hard at this time, while the soil and the rock are very soft, it will appear that the sediment particles squeeze both sides of the rock and soils. Because the rock containing fractures and cracks is soft, the fractures and cracks can be deformed by the force of the hard particles. As shown in Fig. 1, the crack and the fracture are squeezed and get deformed by the hard particles. In this case, stress analysis of the sediment particles in the fissures is necessary.

The geometrical structure of the fissure surface in rock affects the force of particles moving in it. In the geological engineering field, the joint roughness coefficient (*JRC*) is an important parameter that is widely used in the strength evaluation of fractured rocks. Researchers have combined *JRC* and the joint wall compressive strength (*JCS*) to put forward formulas for the relationship between fracture morphology and strength^11–13^, called the *JRC*–*JCS* model. The *JRC*–*JCS* model can be used to determine the peak shear strength of fractured rocks. Based on a large number of model tests and field observations, Barton^14^ proposed the standard roughness grade section. The curve of *JRC* values is divided into 10 sections, the range of which is [0, 20]. In practical applications, the actual structural plane section curve is compared with the standard roughness grade section curve to determine the *JRC* value. Additionally, in this study, the particles moving in the rock fissure are connected with the *JRC* of fissures, which has expanded the application prospects of *JRC*.

The particle in the rock fissure is influenced by the physical and mechanical properties of rock, fluid, fissure and particle, and its force is complex. The static analysis is one of the most important research methods. The static analysis method is widely used in obtaining the stress and stain distribution and structure analysis. For example, Merazi, *et al*.^15^ successfully established a new hyperbolic shear deformation plate theory for the static analysis of a functionally graded material (FGM) plate based on neutral surface position. The CPT-based p-y analysis method^16^ for offshore mono-piles embedded in sands under the conditions of static and cyclic loading conditions is a static analysis with some functions. The static analysis of variable thickness of two-directional functionally graded porous materials is given by Rad, *et al*.^17^. Lopez-Guerra, *et al*.^18^ calculated the standard viscoelastic responses with multiple retardation times through the analysis of static force spectroscopy AFM data. The static analysis in the above studies mainly aims at the conventional structures or special mechanical materials, but the static analysis is not involved in the particles in the rock fissure.

In this study, the static analysis of hard spherical particles in a rock fissure is carried out to obtain the resultant force of the rock fissure on both sides. The resultant force along the fissure is calculated, and the relationship between the resultant force and the *JRC* is established. The influence of the fissure width and the angle between the two sides of rock surface on the resultant force is further studied. It is helpful to understand and solve the plugging and dredge the dam bottom fracture. Next, the sediment and sand are used to plug the rock fracture against leakage. The study in this aspect can provide reference to further developments related to fissure expansion^19^, fissure matrix porosity for the groundwater dual porosity model connecting overlapping continuous conduits network^20^, the permeability distribution affected by fracture^21^, Darcy–Brinkman flow model in narrow crevices^22^, and the stress analysis of the migrating underground sediment within the rock and soil. In hydraulic engineering, it can be applied to the understanding of the dam sediment leak stoppage, dam stability^23^ and groundwater fissure channel unclogging.

## Method and Analysis

The sediment is bound by two sides of the fissure walls, and the other sides (partially from the other sides as well) are free. Therefore, mechanical analysis is necessary in the case of a hard particle moving in a soft rock fracture.

**Figure 2:**
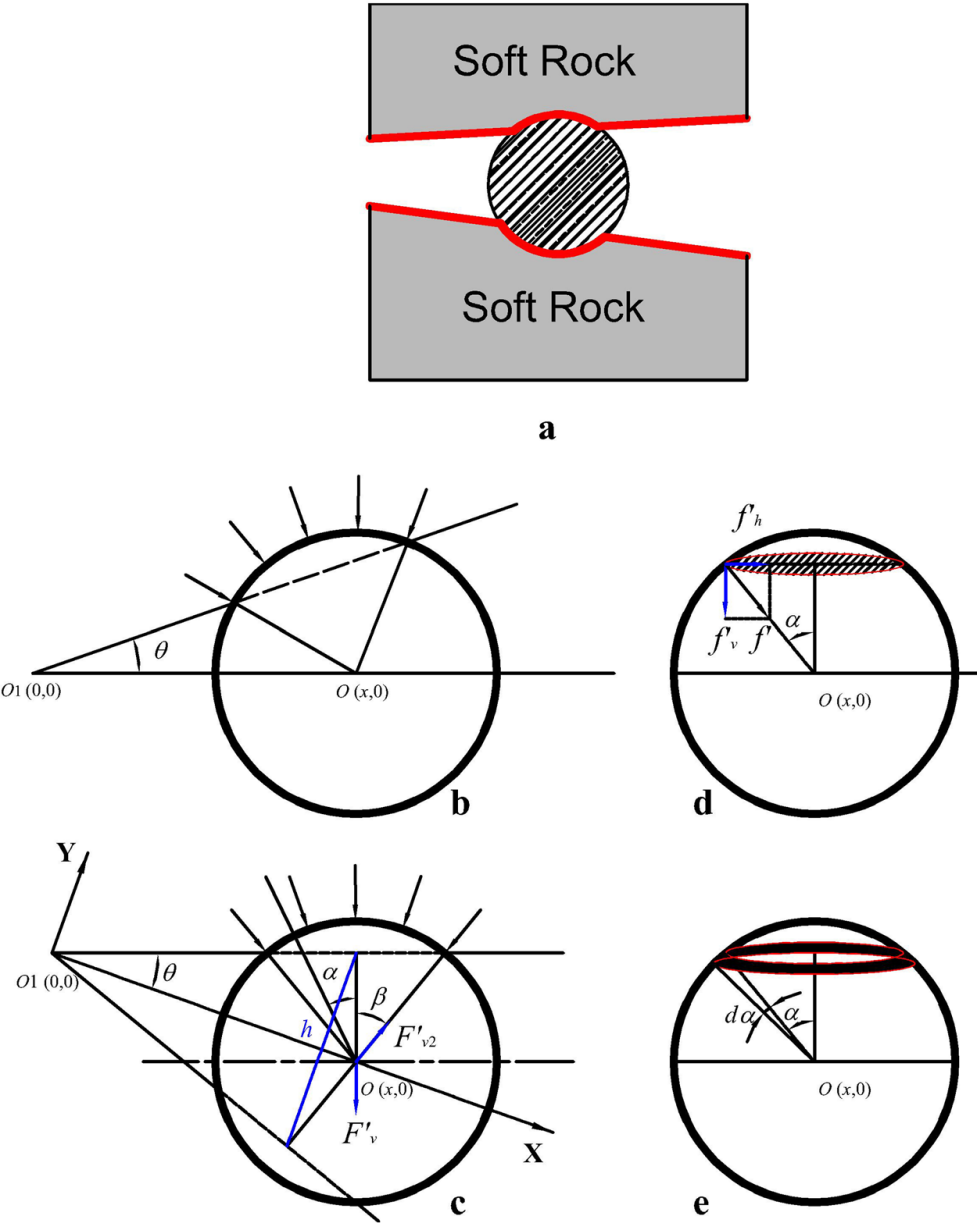
Hard particle squeezes the soft fracture and mechanical analysis. (**a**) Hard particle squeezed by the soft rock from the two sides, which is shown in Fig. 1d. (**b**) Pressure on hard particle from the soft rock. (**c**) After rotating b, the top rock side is horizontal. (**d**) Pressure and force on round surface profiled the ball by horizontal section. (**e**) Pressure and force on ring belt’ on ball, the black shaded area is the ring belt on ball. *h* is the fissure width.

When a hard particle moves in a soft rock fracture, the rock wall deforms (as shown in Figs 1d and 2a), while the hard particle cannot deform or shows very little deformation that can be ignored. In Fig. 2a, the hard particle is pressed by the up and down rock wall. Figure 2b is the cross-section of Fig. 2a. Figure 2b is rotated by an angle into Fig. 2c, in which the fracture up flat is the horizontal plane. Figure 2d is the same as Fig. 2c. The black belt in Fig. 2e is the plane in which the force is same size. *F′*_*v*_ is the resultant force of all *f′*_*v*_. from the upper soft rock plate. *F′*_*v*2_ is the other resultant force from the upper soft rock plate. *R* is the radius of the particle. *F* is the resultant force of *F′*_*v*_ and *F′*_*v*2_.

In Fig. 2, *f′* is the pressure of one point, and the direction of the pressure points to the particle center *O*. On the plate, the horizontal component of *f′* is *f′*_*h*_, and the resultant force of all *f′*_*h*_ is 0. *f′*_*v*_ is the vertical component of *f′*.

In Fig. 2d,e,

1 $$f^{\prime} =k(R-\frac{R\,\cos \,\beta }{\cos \,\alpha }),a\in [0,\beta ]$$

where, *k* is the soft rock elastic coefficient, *α* and *β* are the angle in Fig. 2, and *β* is the max value of *α*.

Since $${f^{\prime} }_{v}=f^{\prime} \cos \,\alpha $$:

2 $${f^{\prime} }_{v}=k(R-\frac{R\,\cos \,\beta }{\cos \,\alpha })\cos \,\alpha $$

The black area in Fig. 2e is given by:

$${\rm{\Delta }}area=2\pi {R}^{2}\,\sin \,\alpha d\alpha $$

The force is given by:

3 $$F{\text{'}}_{v}=\int f{\text{'}}_{v}{\rm{\Delta }}area$$

According to Eqs (1) and (2), Eq. (3) is changed into:

4 $${F^{\prime} }_{v}=\pi k{R}^{3}({\rm{1}}+{\cos }^{2}\beta -2\,\cos \,\beta )$$

Given that α ∈ [0, *β*], *F′*_*v*_ = *F′*_*v*2_ and cos*β* = *x* sin*θ*/*R* in Fig. 2c, Eq. (4) is:

5 $${F^{\prime} }_{v}=\pi kR{(R-x\sin \theta )}^{2}$$

From the resultant force of *F′*_*v*_ and *F′*_*v*2_ in Fig. 2c and Eq. (5), the hard particle joint force is:

6 $$F=2\pi kR{(R-x\sin \theta )}^{2}\,\sin \,\theta $$

The fissure width obtained from Fig. 2 is:

7 $$h=2x\,\sin \,\theta \cdot \,\cos \,\theta $$

## Impact of a Hard Particle in a Soft Fracture

From Eqs (6) and (7), 

$$F=2\pi kR{(R-\frac{h}{2\cos \theta })}^{2}\,\sin \,\theta $$

Thus, the larger *h* is, the smaller *F* is when *R* > *h*/(2 cos*θ*), that is, when the diameter of the hard particle is more than the fissure width.

From Eq. (6), the derivative of *F* with respect to *θ* is,

8 $$\frac{F{\rm{^{\prime} }}|{}_{\theta }}{2\pi kR}=\,\cos (\theta )\times [R-x\times \,\sin (\theta )]\times [R-3x\times \,\sin (\theta )]$$

where *R*-*x* sin*θ* > 0, so sin*θ* < *R*/*x*.

Make Eq. (8) = 0, then

$$\sin (\theta )=\frac{R}{{\rm{3}}x}$$

where sin*θ* = *R*/*x*, *F*′|_*θ*_ = 0, *F* = 0.

From Eqs (6) and (7),

9 $$F=2\pi kR{(R-\frac{hx}{\sqrt{2{x}^{2}+2x\sqrt{{x}^{2}-{h}^{2}}}})}^{2}\frac{h}{\sqrt{2{x}^{2}+2x\sqrt{{x}^{2}-{h}^{2}}}}$$

To further study the mechanics of a hard particle in a soft fracture, an example is considered. The diameter of the hard particle in Shahi and Kuru’s^24^ research is [0.35 mm, 1.18 mm]. According to Eq. (6), in this research, the diameter of the hard particle is chosen as [0.36 mm, 1.18 mm], that is, *R* ∈ [0.18 mm, 0.59 mm]. The fracture is very long, and the two sidewalls are nearly parallel.

As we know, if two walls are parallel, the angle between the two walls is zero. The fissure width is small, and the distance between two sidewalls of fissure is small. Therefore, the two sidewalls are nearly parallel. *θ* is very small. *x* = *R*cos*β*/sin*θ* = *C*/sin*θ*, where *C* is a constant. *x* is very large from the above formula and *θ* is very small.

**Figure 3:**
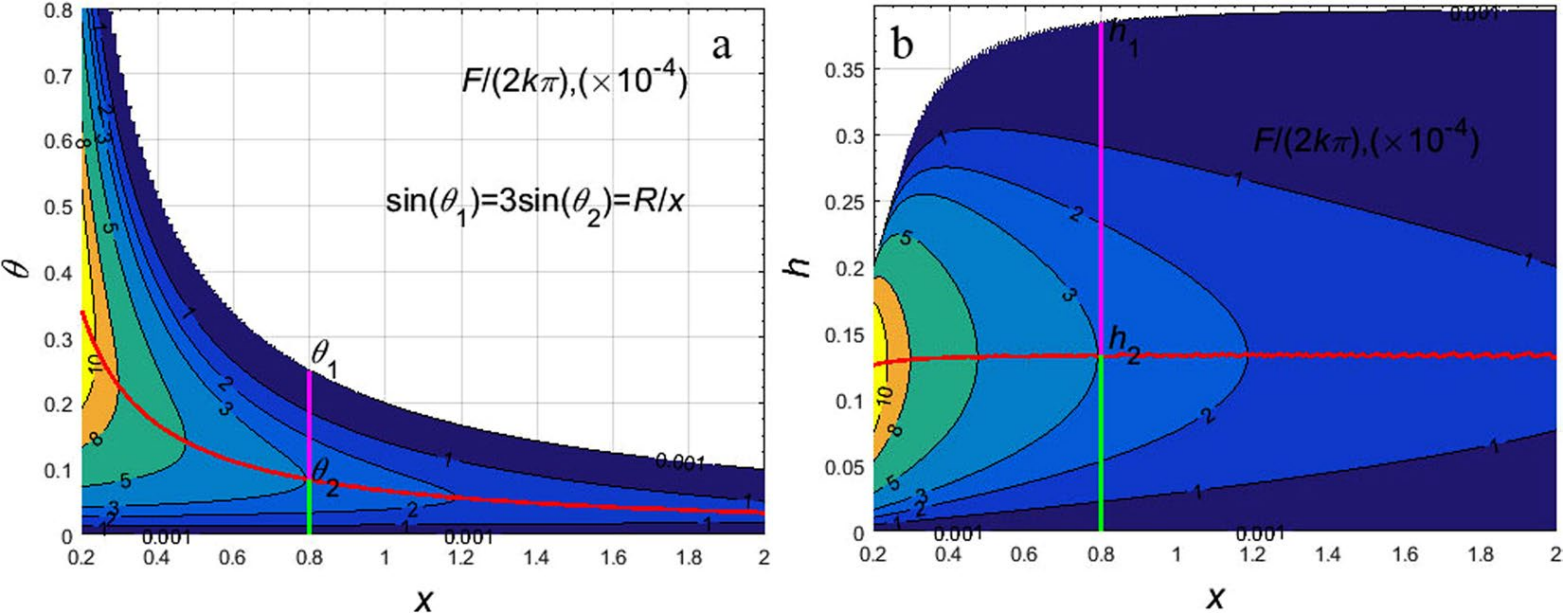
Relationship between the total force and the fissure angle, width and *x*-coordinate of the center of the sphere. (**a**) Relationship between the total force and the fissure angle and *x*-coordinate of the center of the sphere. (**b**) Relationship between the total force and the fissure width/*x*-coordinate of the center of the sphere. The white area in the upper right corner in (**a**) is sin*θ* > *R*/*x*. The red line in (**a,b**) is the maximum points of *F* when *x* is constant and *θ* can change. The green line in Figure **a** is *θ* from 0 to *θ*_2_. The magenta line in (**a**) is *θ* from *θ*_2_ to *θ*_1_. The white area in the upper left corner in (**b**) is sin*θ* > *R*/*x*. The red line in (**b**) is the maximum points of *F* when *x* is constant and *h* can change. The green line in (**b**) is *h*_2_ from 0 to 2 *R*(9*x*^2^ − *R*^2^)^0.5^/(9*x*). The magenta line in (**b**) is *h*_1_ from 2 *R*(9*x*^2^ − *R*^2^)^0.5^/(9*x*) to 2 *R*(*x*^2^ − *R*^2^)^0.5^/*x*. The unit of *F* is N, *k′*s unit is N/mm^3^, *x*’s unit is mm, and *θ*’s unit is rad, and *R* = 0.2 mm in (**a,b**).

From Fig. 1c, the fracture and crack schematic diagram shows bending and twists. Figure 1d is a part of the whole *x* length. *x* of rock fissure is not the real fissure length, but it indicates the relationship between the angle and length. The real fissure is not the whole of the *x* length, but the part of the *x* long fissure. *R* must be larger than *x* × sin(*θ*), so *x* < *R/sin*(*θ*). Therefore, this research chooses the *x* ∈ (0.2 mm, 2 mm] and *θ* ∈ (0, 0.8]. The case result is presented in Fig. 3.

In Fig. 3a, *F* increases with the increase of *θ* when sin*θ* < *R*/(3*x*) that is Eq. (8) > 0. Whereas when *R*/*x* > sin*θ* > *R*/(3*x*) that is Eq. (8) < 0, *F* increases with the decrease of *θ*.

So the maximum of *F* is 8*πkR*4/(27*x*) when sin*θ* = *R*/(3*x*).

In Fig. 3b, From Eq. (9), the derivative of *F* with respect to *h* is 0. *F* increases with the increase of *h* when *h* ∈ (0, 2 *R*(9*x*^2^ − *R*^2^)^0.5^/(9*x*)]. Whereas *F* decreases with the increase of *h* when *h* ∈ [2 *R*(9*x*^2^ − *R*^2^)^0.5^/(9*x*), 2 *R*(*x*^2^ − *R*^2^)^0.5^/*x*].

So the maximum of *F* is 8*πkR*^4^/(27*x*) when *h* = 2 *R*(9*x*^2^ − *R*^2^)^0.5^/(9*x*). This can be shown in Fig. 3a.

From Fig. 2c and Fig. 3b, the resultant force of the sphere particle is away from the top of the fissure. From Fig. 3a,b and Eq. (6), the increase of *x* will lead to the decrease of the resultant force. The increase in the angle causes the resultant force to increase first and then decrease. The extreme point is at sin*θ* = *R*/(3*x*) and *h* = 2 *R*(9*x*^2^ − *R*^2^)^0.5^/(9*x*). And the angle inside the fissure is not static but it changes.

When the fissure tip is consistent with the movement direction of the particle, the movement can be called forward movement, and this angle can be called a positive angle. Similarly, negative movement and negative angle can be defined. When the particles move in the fissures driven by groundwater, they encounter a positive angle and the particles are squeezed by the fissure surfaces, so that the particles are subjected to resistance. When negative angles are encountered, the particles are also squeezed by fissures, at which point the particles accelerate their movement.

From Fig. 3a,b and Eq. (6), the resultant force decreases with the increase of *x*.

**Figure 4:**
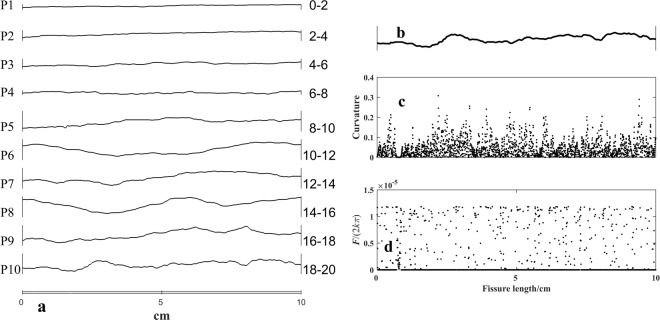
Fracture profile and the fissure, the fissure’s curvature and the hard particle total force from the fissure. (**a**) Ten fracture profiles from Barton and Choubey^14^ and Tse and Cruden^25^. P1, P2, P3…., P10 are the fissure profile’s No. The right numbers, 0–2, 2–4, 4–6, ……, 18–20 are *JRC*. (**b**) The tenth fracture profile, P10 in a. (**c**) The fissure’s curvature of the tenth profile. (**d**) The hard particle total force from the fissure. *F* = 0, when sin*θ* > *R*/*x* = 0.01. The quantity of points is less than that in Figure (**c**). The unit of *F* is N, *k*’s unit is N/mm^3^, *x*’s unit is mm, *θ*’s unit is rad. The mean of the curvature (**c**) is 0.0461. The mean of *F*/(2*kπ*) (**d**) is 1.27 × 10^−6^. The fissure’s *JRC* is 18–20.

The particle’s mechanics are affected by the roughness of the fissure, so we need to consider the roughness to connect with the particle’s mechanics. There are some cracks when the rock cracks. The crack can be treated as the joint of the two side rocks. All the ten typical roughness profiles (as shown in Fig. 4a) in references^14,25^ are chosen as the fissure case to calculate. This research chooses the curvature of the fissure curve as *θ*. The *θ* and curvature of the fissure curve is:

10 $$\theta =\frac{|y^{\prime\prime} |}{{(1+{(y^{\prime} )}^{2})}^{\frac{3}{2}}}$$

The fissure width increases with the curvature of the fissure curve. When *θ* is very small, *h* = 2*xθ*. *x* = 20 mm, *h* can be treated as 20 × sin(2*θ*). According to *R* ∈ [0.18 mm, 0.59 mm], we choose *R* = 0.2 mm. When *R*-*x*sin(*θ*) > 0, the particle is pressed. The particle is free and *F* = 0 when *R*-*x*sin(*θ*) < 0.

The ten fissure profiles, curvature and force results are presented in Fig. 4. Figure 4a shows the ten fracture profiles. Along the tenth fissure (as shown in Fig. 4b), the fissure’s curvature and the hard particle total force are shown in Figs 4c,d.

The mean of curvature, fissure width and *F*/(2*kπ*) of the ten fissures are listed in Table 1. The curvature mean is the average of all the curvatures along the fissure profile curve, based on Eq. (10). The fissure width is the result of Eq. (7), when *x* = 20 mm. The scale of maximum value *θ* is [0.0337, 0.443]. cos*θ* is [0.999,0.999]. *F*/(2*kπ*) is obtained according to Eq. (6) when the *x* < *R*/sin(*θ*). *F*/(2*kπ*) mean is the average value of all results when the *x* < *R*/sin(*θ*). While *x* > *R*/sin(*θ*), according to Eq. (6), the result of *F*/(2*kπ*) is not considered.

**Table 1:** Curvature, fissure width and *F*/(2*kπ*) mean*.

Fissure No.	*JRC*	Curvature Mean $\bar{\theta }$	Fissure width mean $\bar{h}$ (mm)	*F*/(2*kπ*) Mean
1	0–2	5.44 × 10^−3^	0.218	5.94 × 10^−6^
2	2–4	6.82 × 10^−3^	0.273	5.44 × 10^−6^
3	4–6	8.11 × 10^−3^	0.324	4.86 × 10^−6^
4	6–8	8.87 × 10^−3^	0.355	4.61 × 10^−6^
5	8–10	1.31 × 10^−2^	0.522	3.32 × 10^−6^
6	10–12	1.57 × 10^−2^	0.628	2.95 × 10^−6^
7	12–14	2.33 × 10^−2^	0.930	2.00 × 10^−6^
8	14–16	3.08 × 10^−2^	1.230	1.71 × 10^−6^
9	16–18	3.59 × 10^−2^	1.430	1.64 × 10^−6^
10	18–20	4.61 × 10^−2^	1.830	1.27 × 10^−6^

*The Fissure width mean $\bar{h}$ is the average of *h* is the average of all of *h*, including the *h* > 2 *R*(*x*^2^ − *R*^2^)^0.5^. The average of *F* is the average of all of *F* ≠ 0.

From Fig. 4a, the ten profiles show more bending as *JRC* increases. There are more turning and bending points on the higher *JRC* profiles. P1 is the smoothest and has the smallest *JRC* value, [0, 2]. P10 is the most tortuous with the largest *JRC* value [18, 20]. Figure 4c shows the curvature is different along the profile curve. The more the curvature is, the more the curve turns and bends. The more concentrated the turning points are, the more concentrated the large curvature values are. The curvature is 0 at smooth points. *F*/(2*kπ*) changes with the curvature (as shown in Fig. 4d). *F*/(2*kπ*) = 0 when the curvature is 0.

The resultant force *F* accelerates or decelerates the movement of the particle in the fissure. When the fracture dipping direction is consistent with the particle movement direction, *F* decelerates the particle movement. When the fracture dipping direction is against the particle movement direction, *F* accelerates the particle movement. The direction of the resultant force *F* is always opposite to the direction of the tip of the fissure.

**Figure 5:**
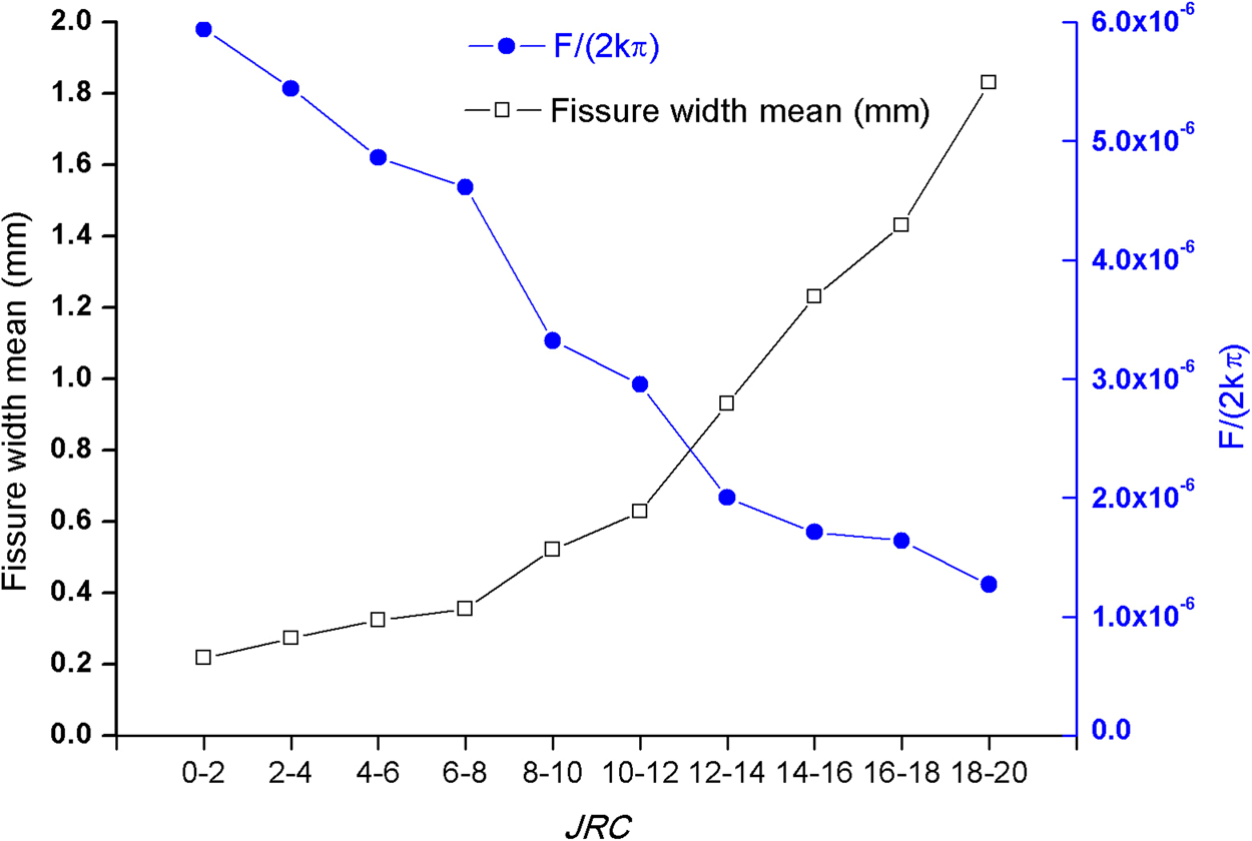
Relationship between *JRC*, Fissure width and *F*/(2*kπ*). *F*/(2*kπ*)’s Mean and Variance in Table 1 are calculated according to *F*/(2*kπ*) ≠ 0. The fissure width and *F*/(2*kπ*) means with *JRC* are plotted in Fig. 5 according to the Table 1 results.

From Table 1 and Fig. 5, it can be seen that the sequence number increases, *JRC* increases, curvature increases, fissure width mean (mm) increases, and *F*/(2*kπ*) decreases. The serial number decreases, *JRC* decreases, curvature decreases, and fissure width mean (mm) and *F*/(2*kπ*) increase.

## Discussion

In Fig. 3, the particle force *F* is affected by *θ* and *x*. From Eq. (6), the resultant force obviously decreases with the increase of *x*. As the width of the fissure *h* is 2*x*sin*θ*·cos*θ*, the particle stress *F* is affected by the angle *θ* and the fissure width *h*. As shown in Table 1 and Fig. 5, there are two main factors that affect the size of particles: the angle between the two sides of the gap and the width of the seam *h*.

### Effect of angle *θ* on force *F*

**Figure 6:**
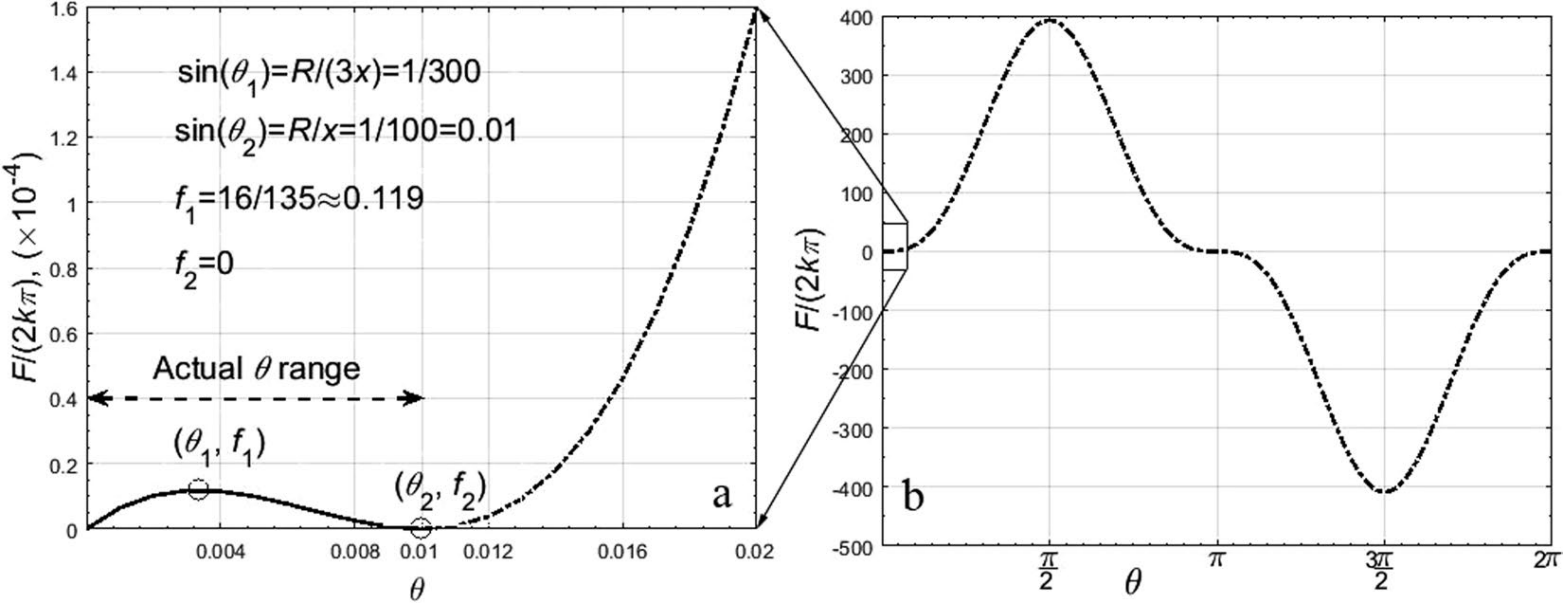
Relationship between *θ* and *F R* = 0.2 mm, *x* = 20 mm. The point (*θ*_1_, *f*_1_) is the maximum point of *F* when sin*θ* = 1/300. The point (*θ*_2_, *f*_2_) is the minimum point of *F* when sin*θ* = 0.01. 0.01 > sin*θ* > 1/300.

From Eq. (6), for a constant *x*, varying *θ*, *R* = 0.2 mm, and *x* = 20 mm, according to the formula (6), the relationship curve between *F*/(2*kπ*) and *θ* is drawn in Fig. 6. Figure 6a is the partial enlargement of Fig. 6b.

In Fig. 6a, *F* increases with the increase of *θ* when sin*θ* < 1/300 that is Eq. (8) > 0. Whereas when 1/100 > sin*θ* > 1/300 that is Eq. (8) < 0, *F* increases with the decrease of *θ*. So the maximum of *F* is 32*πk*/135 when sin*θ* = 1/300. *F* increases first and then decrease with the increase of *θ*.

**Figure 7:**
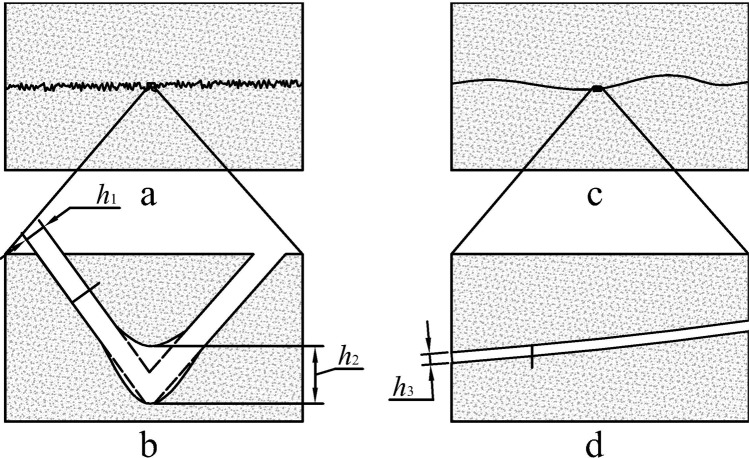
Fissure width of different roughness rock. (**a**) The high *JRC* rock fissure. *h*_1_ and *h*_2_ is the fissure width on a smooth surface and the turning point of the fissure. (**b**) The local enlarged drawing of the high *JRC* rock fissure. (**c**) The low *JRC* rock fissure. (**d**) The local enlarged drawing of the low *JRC* rock fissure, where *h*_3_ is the fissure width. *h*_2_ > *h*_1_ > *h*_3_. *h*_1_, *h*_2_, *h*_3_ are measured perpendicular to the fracture walls.

The influence of the angle *θ* on the force *F* can also be analyzed from the microscopic morphology of the fissure. The microscopic morphology of the rock fissure is plotted in Fig. 7.

Figure 7a is a complex rock section. Figure 7b is a partial enlarged view of Fig. 7a. Figure 7c is a smooth section of the rock, and Fig. 7d is a partial enlarged view of Fig. 7c. Among them, the *JRC* of the rock fissure in Fig. 7a is larger than the *JRC* of the rock in Fig. 7c.

In Figs 4a and 7a,c, the larger the *JRC* is, the coarser the section is, the more turning points the surface has (as in Fig. 7a), the larger the turning degree is, and the larger the angle between the fissure two sides is (as in Fig. 7b). By contrast, the smaller the *JRC* is, the smoother the section is, the fewer turning points the surface has (as shown in Fig. 7c), the smaller the turning degree is, and the smaller the angle between the fissure two sides (Fig. 7d).

### Effect of the fissure average width on the average force

In terms of a stress point of the particle on the fissure profile, when *θ* is a constant, the larger the fissure width *h* (*h* = 2*x*sin*θ·*cos*θ*) is, the larger the *x* is. At same time, the smaller the value of *R*-*x*sin*θ*, the smaller the force of the Eq. (6) is, as shown in Fig. 3.

The turning point of the rock section is greatly increased in Fig. 7a. Many rock debris are mixed in the fissure. Because the fissure is composed of two surfaces instead of two curves, a small rock particle can support a larger gap on both sides of the rock. At the same time, the rock surfaces on both sides of the section are easily misplaced, so the fracture surfaces on both sides do not have very good contact. The larger the distance between the rocks on both sides is, the larger the fissure width *h*_1_ (as shown in Fig. 7b) is. Therefore, *h*_1_ > *h*_3_ (as shown in Fig. 7d) and *F*_1_ < *F*_3_. By contrast, the turning point of the rock section is decreased and the cross section is smooth in Fig. 7c. The rock faces on both sides of the section can easily be placed in close contact. Because of the few points on the fracture surface, the debris in the fissure is minimal. Therefore, the rock space on both sides is smaller and the fissure width is smaller (as shown in Fig. 7d).

Violay, *et al*.^26^ discovered that the rock’s brittle behavior increases the porosity. Violay, *et al*.^26^ concluded that a rock with larger *JRC* is more brittle. The rock mass with higher porosity indicates that the fissure has a larger width and is more complex, and there are more fissures. Thus, we can conclude that the fissure having a larger *JRC* has a larger width and is more complex. Eqs (10) and (7) are reasonable. This research result is consistent with the discovery made in Violay, *et al*.^26^.

Given that the sharp point at the turning point is easily broken, the broken rock at the turning point results in more space, so *h*_2_ > *h*_1_ and *F*_2_ < *F*_1_.

Thus, the influence of the fissure width *h* on the resultant force *F* is suitably explained:

In Fig. 3b, *F* increases first and then decrease with the increase of *h* when *x* is constant. *h* ∈ (0, 2 *R*(*x*^2^ − *R*^2^)^0.5^/*x*]. When *h* > 2 *R*(*x*^2^ − *R*^2^)^0.5^/*x*, *F* = 0.

In Table 1 and Fig. 5, the average of *F* decrease with the increase of the average of *h*. The average of *F* is the average of all of *F *≠ 0. The average of *h* is the average of all of *h*, including the *h* > 2 *R*(*x*^2^ − *R*^2^)^0.5^. For example, there are three points (*h*, *F*) on Fissure 1 that are (*R*(*x*^2^ − *R*^2^)^0.5^/*x*, *F*_1_), (2 *R*(*x*^2^ − *R*^2^)^0.5^/*x*, 0) and (3 *R*(*x*^2^ − *R*^2^)^0.5^/*x*, 0). The average of all of *F* is *F*_1_, and the average of all of *h* is 2 *R*(*x*^2^ − *R*^2^)^0.5^/*x*. There are three points (*h*, *F*) on Fissure 2 that are (2 *R*(*x*^2^ − *R*^2^)^0.5^/*x*, 0), (3 *R*(*x*^2^ − *R*^2^)^0.5^/*x*, 0) and (4 *R*(*x*^2^ − *R*^2^)^0.5^/*x*, 0). The average of all of *F* is 0, and the average of all of *h* is 3 *R*(*x*^2^ − *R*^2^)^0.5^/*x*. So *F*_1_ > 0, 2 *R*(*x*^2^ − *R*^2^)^0.5^/*x* < 3 *R*(*x*^2^ − *R*^2^)^0.5^/*x*.

So the rules shown in Fig. 3b and Table 1/Fig. 5 seem to be different. In fact, the above explanation well explains this different. *x* is constant in Fig. 3 and averaging the width *h* and angle *θ* in Table 1 and Fig. 5 is the key issue.

From the result in Fig. 4d, we can see that the total force from the two sides of the fissure changes along the fissure. The quantity of the force is connected with the fissure open angle and the fissure width.

This research combines the fissure open angle and the width to analyze the resultant force of a hard particle under both sides of the fissure. Along the fissure, the size of the resultant force is not regular and shows some randomness and uncertainty. This agrees with the general experimental observation because the fissure is caused by a fracture, and the material is heterogeneous. The expansion of the crack is very random, and the fissure width is uncertain. This is why the *JRC*^27,28^ is investigated in several studies. The trajectory shape, the surface roughness and the open angle of fissures play important roles in the mechanical action of particles.

## Conclusion

In this study, the hard particle static force in a soft rock fracture is analyzed. The total force, as given in Eq. (6), is established. The power source is the groundwater seepage. The water motion causes the particle to be squeezed by the top and bottom rock plates. The results lead to a better understanding of the transporting of a nondeformable solid in a rock fissure. The analysis process provides a reference for the calculation of the transporting of underground sand through the fissure of rock-soil body at irrigation works.

In terms of a point on the fissure and a constant *x*, there are a fixed *θ*, *h*, *F*. *F* increases first and then decrease with the increase of *θ* and *h*. The average of *h* and *θ* have the similar effect on the average of *F*. The extreme point is sin*θ* = *R*/(3*x*), and *h* = 2 *R*(9*x*^2^ − *R*^2^)^0.5^/(9*x*). At the same time, the maximum of *F* is 8*πkR*^4^/(27*x*).

It is found that the bigger *JRC* of fissure is, the larger the average of fissure open angle *θ* is and the larger the average width *h* is. As the average width *h* or the average angle *θ* increases, the average resultant force of *F* decreases. The average of *h* and *θ* have the similar effect on the average of *F*.

Along the fissure, the size of the resultant force is not regular and shows some randomness and uncertainty. The mechanical effect of fissures on the particles is mainly affected by the trajectory morphology, surface roughness, and the open angle of the fissures. The measurement of these factors is an important part of the future study of the forces driven by subsurface fluids in fractures. The measurement of these factors is very important, especially in future mechanical studies on the particle movement driven by underground fluid in rock fissures.

The study can be used in these studies: Darcy–Brinkman flow model in narrow crevices^29,30^, clay fill in embankment fissures^31^, permeability of noncohesive soils^32^, the groundwater hydrology model^33^, and others.

The open angle and the width of rock fissures are calculated based on the fissure profile curvature, which conforms to a certain actual situation. In this research, the connection between the direction of the resultant force *F* and the fissure *JRC* is not considered because of the considerable difficulty in measuring the fissure. More detailed and more accurate calculation and measurement need to be carried out by technical researchers in the future.
